# Diversity of *Aedes* Mosquito Breeding Sites and the Epidemic Risk of Arboviral Diseases in Benin

**DOI:** 10.3390/insects16121215

**Published:** 2025-11-28

**Authors:** Germain Gil Padonou, Isidore Hoyochi, Arthur Sovi, Alphonse Keller Konkon, David Mahouton Zoungbédji, Albert Sourou Salako, Constantin Jésukèdè Adoha, Arsène Fassinou, Come Z. Koukpo, Saïd Chitou, Udoka Nwangwu, Anges Yadouleton, Lamine Baba-Moussa, Martin Codjo Akogbéto

**Affiliations:** 1 Centre de Recherche Entomologique de Cotonou, Cotonou 06 BP 2604, Benin; pagergil@yahoo.fr (G.G.P.); konkonkelleralphonse@gmail.com (A.K.K.); davidzoungbedji91@gmail.com (D.M.Z.); adohaj.constantin@yahoo.fr (C.J.A.); arsenf88@yahoo.fr (A.F.); zkoukpo@gmail.com (C.Z.K.); saochitou@gmail.com (S.C.); anges33@yahoo.fr (A.Y.); akogbetom@yahoo.fr (M.C.A.); 2 Faculté des Sciences et Techniques, Université d’Abomey-Calavi, Calavi 01 BP 4521, Benin; laminesaid@yahoo.fr; 3 Faculté d’Agronomie, Université de Parakou, Parakou BP 123, Benin; 4 Department of Diseases Control, The London School of Hygiene and Tropical Medicine, London WC1E 7HT, UK; 5 Département de Médecine Vétérinaire, Institut Supérieur des Sciences et de Médecine Vétérinaire (ISSMV) de Dalaba, Dalaba BP 2201, Guinea; albertsourousalako@yahoo.fr; 6 National Arbovirus and Vectors Research Centre (NAVRC), Enugu 400102, Nigeria; nwangwuudoka@gmail.com; 7 Ecole Normale Supérieure de Natitingou, Université Nationale des Sciences, Technologies, Ingénierie et Mathématiques, Goho, Abomey BP 2282, Benin; 8 Laboratoire de Biologie et de Typage Moléculaire en Microbiologie, Département de Biochimie et de Biologie Cellulaire, Université d’Abomey-Calavi, Calavi 05 BP 1604, Benin

**Keywords:** types of breeding sites, *Aedes*, epidemic risk, arboviral diseases, Benin

## Abstract

*Aedes* mosquitoes are major vectors responsible for the transmission of arboviral diseases. This study aimed to identify the types of breeding sites colonized by *Aedes* mosquitoes and to assess the epidemic risk of arboviral diseases across communes in Benin. From January to November 2024, mosquito larvae were collected in nine study communes. All water-holding containers encountered were inspected for larvae and pupae. Samples of larvae and pupae were collected, pooled by breeding site type, and reared to adulthood for species identification. The frequency of different breeding site types was recorded. Epidemic risk was assessed using the House, Container and Breteau indices. *Aedes aegypti* (70.7%) was the most abundant species, followed by *Culex quinquefasciatus* (25.3%). Breeding sites were mainly artificial and included domestic containers (buckets, jars, drums, cans, cisterns, water troughs), tyres, and discarded containers (tin cans). The House, Container and Breteau indices indicated a high epidemic risk of arboviral diseases across the communes. The wide diversity of breeding sites supporting *Aedes* development, coupled with the high epidemic risk indices, highlights the urgent need for integrated vector control strategies, with priority given to identified high-risk areas.

## Simple Summary

Mosquitoes of the *Aedes* genus transmit several arboviral diseases, including dengue fever, chikungunya, and Zika, to humans. In order to better understand the risk of epidemic occurrence, the various mosquito breeding sites were enumerated in some Benin communes between January and November 2024. All potential breeding sites were inspected both indoors and outdoors for the presence of larvae and pupae of *Aedes* mosquitoes. The results showed that *Ae. aegypti* was the most abundant mosquito species, breeding mainly in artificial water containers such as buckets, jars, cans, used tyres, and discarded containers. The Stegomyia indices indicated there was a high epidemic risk in all study communes. These findings emphasize the urgent need to establish a management plan for mosquito breeding sites. This is required to protect populations from *Aedes*-borne diseases and limit their health burden.

## 1. Background

Mosquitoes are major vectors of several human diseases, including malaria, dengue fever, lymphatic filariasis and others [1]. They are holometabolous insects whose life cycle includes a mandatory aquatic phase [2]. This phase begins with egg laying, followed by the hatching of larvae that metamorphose into pupae within aquatic habitats [2]. The availability, diversity and distribution of these larval habitats are key factors influencing mosquito proliferation.

Mosquito-borne diseases continue to undermine global public health, particularly in Africa [3]. In 2023, more than 5 million cases of dengue fever and 5000 deaths were reported in over 80 countries worldwide [4]. In Africa alone, 171,991 cases of dengue and 753 deaths were documented across several countries [5], including Burkina Faso, Cape Verde, Senegal and Nigeria [6,7,8,9]. This spread of the virus highlights the increased risk of an epidemic on the continent. Given its geographical proximity to Burkina Faso and Nigeria, Benin remains at considerable risk of arbovirus outbreaks. This risk has become more evident in recent years with the detection of dengue cases in 2019, alongside the identification of multiple serotypes of the virus (DENV-1, DENV-3 and DENV-4) in vectors from southern Benin [10,11,12].

Clinically, dengue presents with fever, headache, myalgia, arthralgia, rash, nausea, diarrhea, vomiting and abdominal pain [13]. Its symptoms overlap with malaria, complicating clinical diagnosis and often leading to inappropriate treatment. The dengue virus is primarily transmitted by mosquitoes of the *Aedes* genus, which typically thrive in densely populated urban areas [14,15,16,17,18]. They colonize a wide range of domestic and peri-domestic larval habitats, including plastic containers, barrels, tyres, discarded cans, buckets and jars [5].

Since mosquito larvae depend on aquatic environments, effective management of domestic and peri-domestic breeding sites is a critical strategy for reducing vector density [2,19]. Such management involves eliminating or modifying breeding sites to prevent the emergence of adult mosquitoes [20]. A sound understanding of mosquito ecology, particularly their breeding site preferences, is therefore essential for designing sustainable vector control strategies [15,21]. However, in Benin, few studies have documented the diversity and ecological characteristics of *Aedes* larval habitats, even though this information is crucial for guiding vector control interventions.

Targeted management of breeding sites can significantly reduce *Aedes* mosquito populations and, consequently, minimize the risk of arboviral disease transmission.

This study aimed to identify the types of breeding sites colonized by *Aedes* mosquitoes and to assess the epidemic risk of arboviral disease emergence in Benin.

## 2. Materials and Methods

### 2.1. Study Area

**Figure 1:**
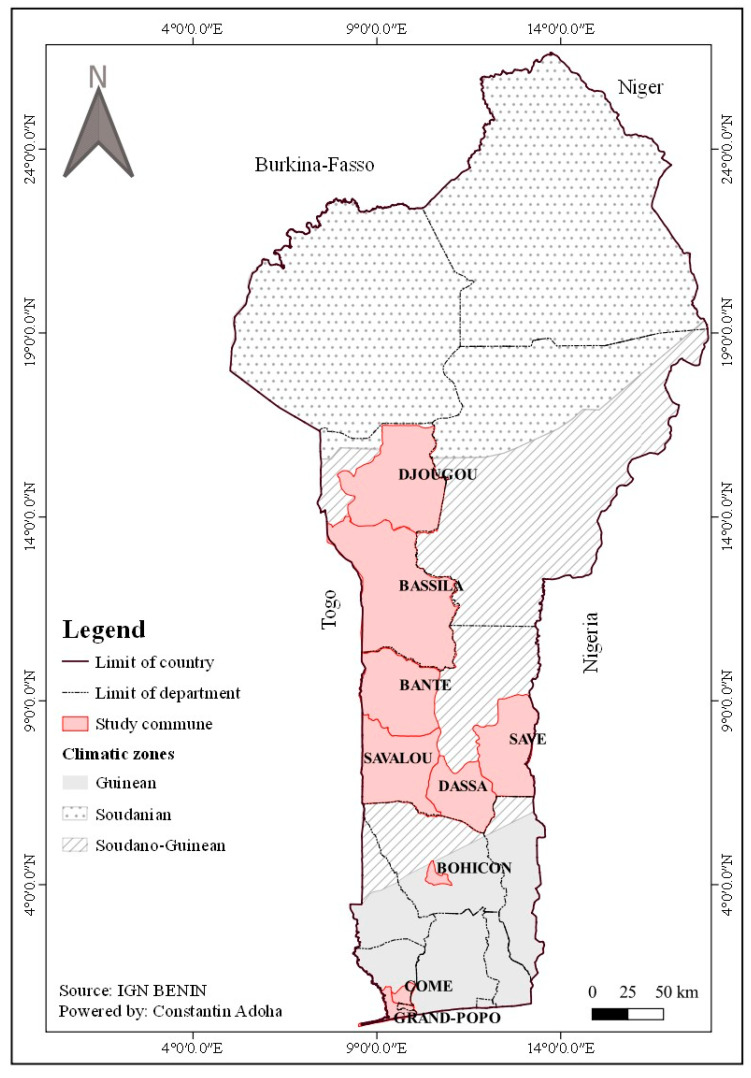
Map of the study area showing the study communes.

The study was conducted in nine communes in Benin, spanning two climatic zones: the Guinean zone and the Sudano-Guinean zone. The Guinean zone is characterized by a sub-equatorial climate with two rainy seasons (April–July and mid-September–November) and two dry seasons (August–mid-September and December–March). Annual rainfall ranges from 900 to 1400 mm, while temperatures generally vary between 25 °C and 30 °C throughout the year [22]. The main economic activities in communes located within this zone (Comé, Grand-Popo and Bohicon) include cultivation of food crops (maize, cassava, cowpeas), market gardening, local crafts, fishing and trade (Figure 1). According to the 2013 General Population and Housing Census (RGPH-4), Comé had an estimated population of 79,989, Bohicon 171,781, and Grand-Popo, 76,597. The Sudano-Guinean zone also experiences a sub-equatorial climate, with a rainy season from May to October and a dry season from November to April. Average annual rainfall ranges between 900 and 1200 mm and temperatures vary from 24 °C to 34 °C, with peaks in March and April [22]. The tropical ferruginous soils are highly suitable for agriculture [23]. Dominant activities in this zone include the cultivation of maize, yams, cassava, peanuts, cotton, rice, cowpeas and cashews, as well as market gardening, livestock farming and trade. The communes of Dassa-Zoumé, Savalou, Savè, Bantè, Bassila and Djougou were covered in this zone (Figure 1). According to the 2013 RGPH-4 data, the populations were 112,122 in Dassa-Zoumé, 144,549 in Savalou, 107,181 in Bantè, 130,091 in Bassila, 267,812 in Djougou and 87,177 in Savè. Across all study communes, domestic water management practices rely on the storage of rainwater in cisterns and jars, along with the conservation of well water in containers such as jerry cans, buckets and drums.

The communes selected for this study were distributed along a south–north transect of Benin to deepen the understanding of the spatial dynamics of *Aedes* populations and to investigate the ecological evolution of these vectors. The selection was also based on epidemiological and geographical considerations. Some of the communes are located near borders with Nigeria and Burkina Faso, two countries where dengue transmission has already been documented, thus posing a potential risk of virus introduction and spread. Moreover, between April and July 2019, several laboratory-confirmed dengue cases, including some fatalities, were reported in Benin, mainly in the departments of Atlantique, Littoral, and Ouémé [10]. Previous studies have further highlighted a high risk of arboviral epidemics and confirmed the presence of *Aedes albopictus* in several southern communes, including Ifangni, Kétou, Sakété, Pobè, Adja-Ouèrè, Adjara, and Avrankou [12].

### 2.2. Contact and Consent of Heads of Households and Villages

Prior to inspecting breeding sites in selected households, consent was obtained from both village leaders and household heads. With the assistance of a community guide, the team from the Centre for Research in Entomology of Cotonou (CREC) explained the purpose of the study and highlighted the health risks associated with mosquito breeding sites in and around households.

### 2.3. Sampling of Immatures of Mosquito

Mosquito larvae and pupae were collected from January to November 2024 in the nine study communes. In each commune, 10–53 households were inspected. This variable number of households inspected per commune was due to several factors, including acceptance by households, availability of residents, weather conditions, and accessibility.

All breeding sites encountered in domestic and peri-domestic environments were inspected and data were recorded using a tablet. Breeding sites were classified into categories, namely domestic containers (buckets, jars, canaries, barrels, cans, flower pots, cisterns, drinking troughs); tyres; discarded containers (plastic bags, tin cans); natural containers (tree holes, sheathing plants); other breeding sites (abandoned cars, mortars, wheelbarrows, fruit shells). All water-holding containers encountered during the survey were examined to determine the presence (positive containers) or absence (negative containers) of mosquito immatures inside and around households. Larvae and pupae were collected from positive containers using a ladle and flashlight. The geographical coordinates of each surveyed household were recorded with a GPS device.

Batches of larvae and pupae were labelled with the date, household number and village name, then transported to the insectary of CREC. They were reared under standard insectary conditions (27 ± 2 °C et 75 ± 5% relative humidity) until adult emergence [24,25]. Emerged adults were morphologically identified using standard taxonomic keys developed by Edwards [26], Huang et al. [27] and Coetzee [28].

### 2.4. Data Analysis and Interpretation

#### 2.4.1. Assessment of the Epidemic Risk of Occurrence of Arboviral Diseases

Entomological indices used to estimate the epidemic risk of arboviral disease transmission were calculated based on breeding sites colonized by *Aedes* larvae and pupae [29]. The following Stegomyia indices were determined to assess epidemiological risk [29]:

- Container Index (CI): percentage of water-holding containers infested with *Aedes* larvae or pupae.
- Breteau Index (BI): number of positive containers per 100 houses inspected.
- House Index (HI): percentage of houses infested with *Aedes* larvae or pupae.

#### 2.4.2. Diversity of Mosquito Breeding Sites

The diversity and spatial distribution of breeding sites were analyzed using QGIS software (version 3.34). GPS coordinates of the breeding sites were recorded and integrated into a geographic information system. Maps were generated to illustrate the spatial distribution of both potential and infested breeding sites, thereby providing a geographical visualization of areas at risk for arboviral diseases.

The proportions of the different breeding site types (infested and non-infested) and different species were compared using a Chi-square test of proportions. Significant differences between values were denoted by different letters (a, b, c). The level of statistical significance was set at *p* < 0.05.

The Stegomyia indices (House Index, Breteau Index and Container Index) were also calculated and interpreted according to WHO standards [30]:

- Container index (CI) = number of breeding sites infested with Aedes larvae and pupae × 100∕number of breeding sites inspected. It is interpreted as follows:
- CI < 3, low epidemic risk;
- 3 ≤ CI ≤ 20, moderate epidemic risk;
- CI > 20, high epidemic risk.
- Breteau index (BI) = number of positive breeding sites found in 100 inspected houses. This index is interpreted as follows:
- BI < 5, low epidemic risk;
- 5 ≤ BI ≤50, moderate epidemic risk;
- If BI > 50, high epidemic risk.
- House index (HI) = number of houses with positive breeding sites for Aedes larvae × 100∕number of houses inspected. The following ranges are used to interpret this index:
- HI < 4, low epidemic risk;
- If 4 ≤ HI ≤ 35, moderate epidemic risk;
- If HI > 35, high epidemic risk.

## 3. Results

### 3.1. Main Mosquito Breeding Sites

**Figure 2:**
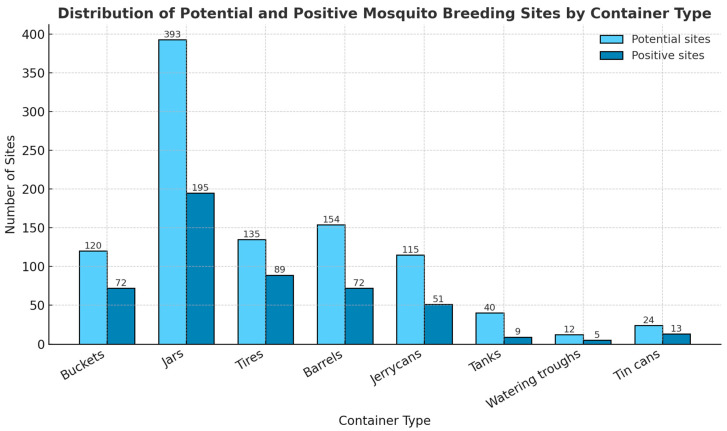
Mosquito breeding sites (potential and positive).

Field surveys revealed a variety of larval habitats, with artificial habitats dominating (Figure 2).

A total of 993 potential breeding sites were inspected during the study, of which 506 (51.0%) were positive for *Aedes* larvae. Among the different types of containers identified, jars were the most common (393 sites), with 195 (49.6%) found positive. Tires represented 135 sites, 89 (65.9%) of which contained larvae, making them one of the most productive container types. Buckets also showed a high level of infestation, with 72 (60.0%) out of 120 positive. Barrels and jerrycans accounted for 154 and 115 sites, respectively, with positivity rates of 46.8% and 44.3%. In contrast, tanks, watering troughs, and tin cans were less frequently encountered (40, 12, and 24 sites, respectively) and exhibited lower positivity rates (22.5%, 41.66% and 54.16%) (Figure 2).

These findings indicate that domestic containers such as buckets, jars, and jerrycans were the most abundant and productive breeding habitats for *Aedes* mosquitoes. This highlights the significant role of household water storage practices and human activities in shaping the breeding dynamics of these vectors in the study area.

### 3.2. Mosquito Species Composition

*Aedes* larvae were found either alone or in sympatry with *Culex* and *Anopheles* larvae in the sampled breeding sites. Overall, seven mosquito species were identified upon adult emergence: *Aedes aegypti*, *Ae. albopictus*, *Ae. vittatus*, *Anopheles gambiae* s.l., *Culex nebulosus*, *Cx. tigripes* and *Cx. quinquefasciatus*, with relative abundances varying by breeding site type (Table 1).

*Aedes aegypti* was the most abundant species, representing 70.7% of the total, followed by *Culex quinquefasciatus* at 25.3%. Other species were present at very low proportions (<2%).

**Figure 3:**
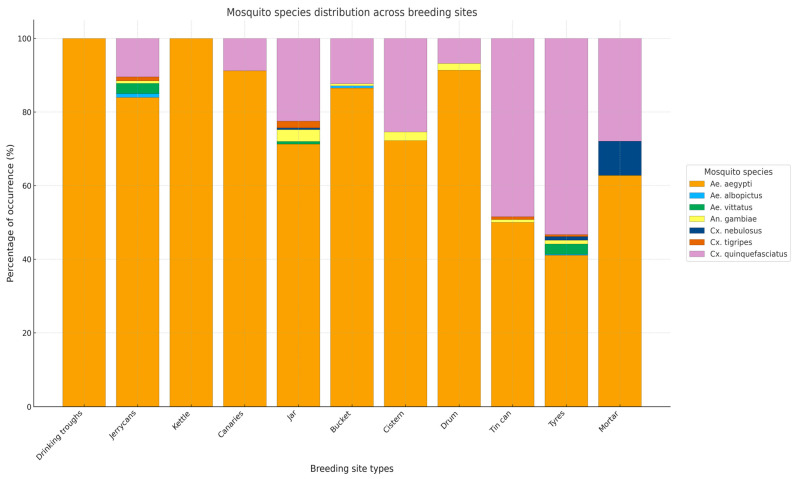
The distribution of species across different types of breeding habitat.

Among the vectors of arboviral diseases, *Ae. aegypti* was detected in all breeding site types, whereas *Ae. albopictus* was only found in jerrycans, tyres and buckets (Table 1, Figure 3). The distribution of species in different types of containers is statistically significant (*p* < 0.0001), confirming that *Ae. aegypti*, *Ae. albopictus* and *Cx. quinquefasciatus* have specific preferences for certain types of breeding sites (Figure 3). *Ae. aegypti* mainly colonises domestic breeding sites such as jerrycans, jars and buckets; *Ae. albopictus* is limited to artificial containers such as tyres, jerrycans, and buckets; and *Cx. quinquefasciatus* prefers tyres, discarded containers, and jars. These results highlight the importance of tailoring vector control interventions to the type of breeding site and dominant species to optimise the effectiveness of arbovirus surveillance and prevention (Table 1, Figure 3).

### 3.3. Diversity and Frequency of Breeding Sites

**Figure 4:**
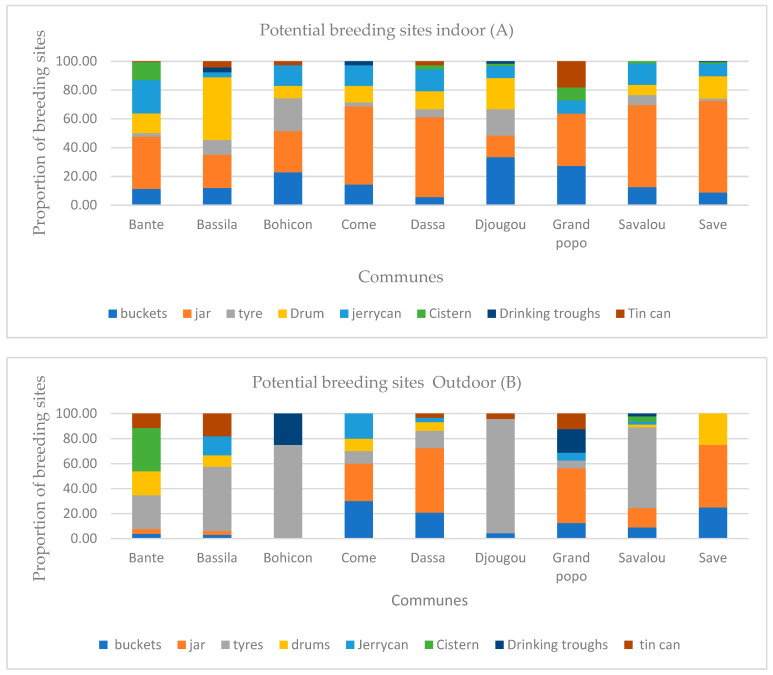
Proportion of potential breeding sites (Indoor (**A**) and Outdoor (**B**)).

**Figure 5:**
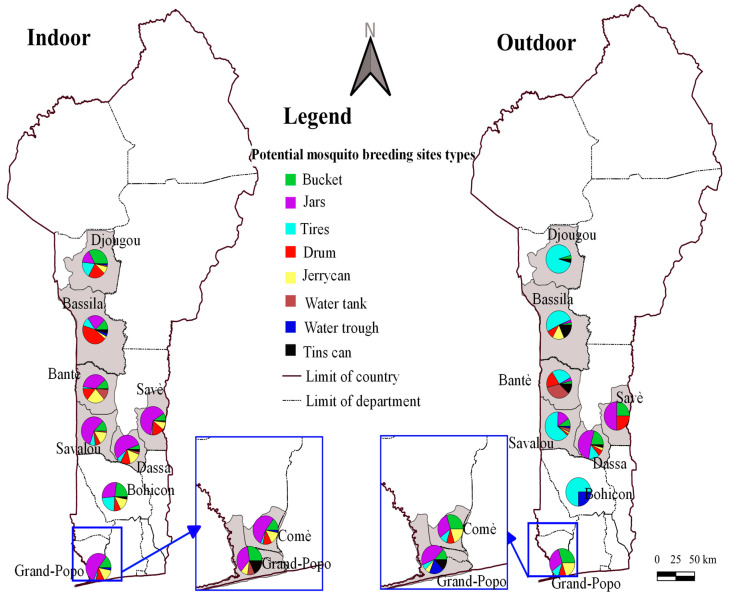
Mapping of the frequency of potential mosquito breeding sites by commune.

**Figure 6:**
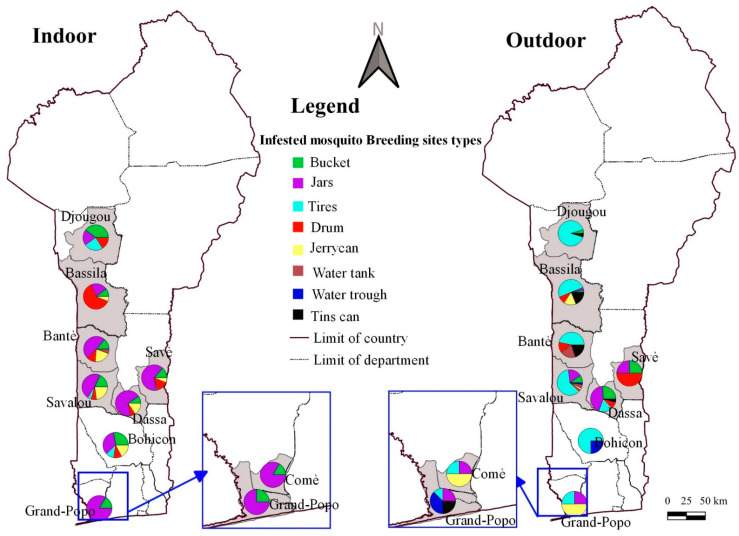
Mapping of the frequency of infested mosquito breeding sites by commune.

Figure 4, Figure 5 and Figure 6 show the frequencies of potential mosquito breeding sites and those infested with *Aedes* larvae, indoors and outdoors, respectively. Overall, breeding sites were diverse and included buckets, jars, drums, jerrycans, cisterns, drinking troughs, tyres and tin cans.

In Bantè, potential breeding sites included jars (36.3% indoors, 3.9% outdoors), jerrycans (23.2% indoors, 0% outdoors), buckets (11.3% indoors, 3.9% outdoors), drums (13.7% indoors, 19.2% outdoors), tyres (2.4% indoors, 26.9% outdoors), cisterns (12.5% indoors, 34.6% outdoors) and tin cans (11.5% outdoors) (Figure 4 and Figure 5). Indoor *Aedes* larvae were most frequently found in jars (48.6%) and jerrycans (20.8%), while outdoors they were common in tyres (46.7%), tanks (20%) and cans (20%). In Bassila, potential breeding sites were buckets (12.0% indoors, 3.0% outdoors), jars (23.1% indoors, 3.0% outdoors), tyres (10.3% indoors, 51.5% outdoors), drums (43.6% indoors, 9.1% outdoors) and tin cans (4.3% indoors, 18.2% outdoors). Indoor *Aedes* larvae were mainly found in drums (62.5%) and jars (20.8%), while outdoors they were prevalent in tyres (50%) and tin cans (18.8%). In Bohicon, potential breeding sites indoors were buckets (22.9%), jars (28.6%), tyres (22.9%), drums (8.6%) and jerrycans (14.3%), compared to tyres (75%) and watering troughs (25%) outdoors. Indoor larvae were most frequent in buckets (27.6%) and jars (34.5%) and outdoors in tyres (75%). In Djougou, potential breeding sites were buckets (33.3% indoors, 4.4% outdoors), jars (15% indoors, 0% outdoors), tyres (18.3% indoors, 91.3% outdoors), drums (21.7% indoors, 0% outdoors) and jerrycans (0% indoors, 4.4% outdoors). Indoor *Aedes* larvae were most common in buckets (40%), while outdoors they were prevalent in tyres (91.9%). In Grand-Popo, potential breeding sites included buckets (27.3% indoors, 12.5% outdoors), jars (36.4% indoors, 43.8% outdoors), tyres (0% indoors, 6.3% outdoors), drinking troughs (0% indoors, 18.8% outdoors) and tin cans (18.2% indoors, 12.5% outdoors). Indoor larvae were found mainly in jars (75%) and buckets (25%) and outdoors in drinking troughs (37.5%) and tin cans (25%). In Savalou, potential breeding sites were buckets (12.5% indoors, 8.9% outdoors), jars (57.0% indoors, 15.6% outdoors), tyres (7.0% indoors, 64.4% outdoors), drums (7.0% indoors, 2.2% outdoors), jerrycans (14.8% indoors, 2.2% outdoors) and cisterns (1.6% indoors, 4.4% outdoors). Indoor larvae were most frequent in jars (47.7%) and jerrycans (22.7%) and outdoors in tyres (61%).

In Comé, potential breeding sites included buckets (14.3% indoors, 30% outdoors), jars (54.3% indoors, 30% outdoors), tyres (2.9% indoors, 10% outdoors), drums (11.4% indoors, 10% outdoors) and jerrycans (14.3% indoors, 20% outdoors). Indoor larvae were most frequent in jars (83.3%) and buckets (16.7%) and outdoors in jerrycans (50%) and tyres (25%).

In Dassa-Zoumé, potential breeding sites were buckets (5.6% indoors, 20.7% outdoors), jars (55.6% indoors, 51.7% outdoors), tyres (5.6% indoors, 13.8% outdoors), barrels (12.5% indoors, 6.9% outdoors), drums (15.3% indoors, 3.4% outdoors) and tin cans (2.8% indoors, 3.4% outdoors). Indoor larvae were most frequent in jars (68.8%) and jerrycans (15.6%) and outdoors in jars (43.5%) and tyres (17.4%). In Savè, potential breeding sites included jars (63.5% indoors, 50% outdoors), buckets (8.8% indoors, 25% outdoors), tyres (5.6% indoors, 13.8% outdoors) and drums (15.5% indoors, 25% outdoors). Indoor larvae were most frequent in jars (64.2%) and drums (14.8%) and outdoors in drums (50%). Overall, artificial containers such as jars, tyres, buckets, drums and cans predominated as major breeding habitats for *Aedes* mosquitoes across all study communes (Figure 6).

### 3.4. Classification of Potential and Infested Mosquito Breeding Sites

**Figure 7:**
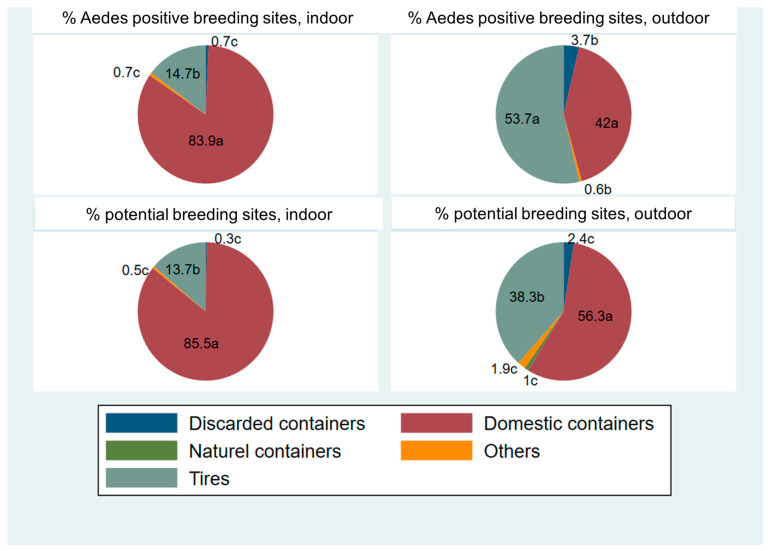
Proportion of mosquito breeding sites (potential and positive) indoors and outdoors. Parts of the same pie chart with the letters a, b, and c are significantly different (*p* < 0.05).

In the study area, household containers proved to be the most abundant habitats, accounting for 85.5% of indoor habitats and 56.3% of outdoor habitats, followed by tyres, which contributed 13.7% and 38.3%, respectively. Discarded containers (0.3% indoors; 2.4% outdoors) and other types of shelters (0.5% indoors; 1.9% outdoors) were observed in significantly lower proportions (Figure 7). The *p*-values below 0.0001 indicate that these differences between breeding site types are highly significant, confirming that domestic containers are significantly more common habitats than tires or any other type of breeding site, both inside and outside houses (Table 2).

A similar trend was observed regarding positive breeding sites containing *Aedes* larvae: Domestic containers remained dominant, accounting for 83.9% of positive breeding sites indoors and 42% outdoors, while tyres accounted for 14.7% and 53.7%, respectively (Figure 6 and Figure 7). Once again, the differences observed were statistically very significant (*p* < 0.0001), reflecting a non-random distribution of larvae according to the type of breeding site (Table 2). These results demonstrate that artificial breeding sites, particularly domestic containers and tyres, play a pivotal role in the proliferation of *Aedes* mosquitoes in the municipalities under study.

Therefore, the high abundance and positivity of domestic containers compared to other breeding sites, as evidenced by extremely low *p*-values (<0.0001), highlight the importance of prioritising these types of breeding sites in vector management and control strategies.

### 3.5. Assessment of the Epidemic Risk of Arboviral Disease Occurrence

Table 3 presents the Stegomyia indices used to estimate the epidemic risk of arboviral outbreaks across the study communes.

Based on the Container Index (CI), all communes exhibited a high epidemic risk, with values ranging from 41.38% in Grand-Popo to 91.67% in Dassa-Zoumé (Table 2). Similarly, the Breteau Index (BI) indicated a high epidemic risk in all communes, with values ranging from 60 in Grand-Popo to 275 in Bohicon.

For the House Index (HI), high epidemic risk was observed in Bantè (54.04%), Bassila (54.29%), Bohicon (66.67%), Comé (90%), Dassa-Zoumé (41.67%), Savalou (50%) *and* Savè (45.28%), whereas moderate risk was recorded in Djougou (27.03%) and Grand-Popo (35%) (Table 2).

Overall, the Stegomyia indices indicate a generally high risk of arboviral disease outbreaks across most of the study communes.

## 4. Discussion

This study revealed a high risk of arboviral epidemics in the surveyed communes, with *Aedes aegypti* emerging as the predominant species, followed by *Culex quinquefasciatus*. The predominance of *Ae. aegypti* aligns with its well-established role as the principal vector of dengue, chikungunya, Zika, and yellow fever viruses in Africa and beyond [31,32,33]. The detection of *Ae. albopictus*, although restricted to drums, buckets, and tyres, indicates a strong ecological preference for artificial containers, consistent with findings from Cameroon and Central Africa [34]. Regional reports from Côte d’Ivoire, Nigeria, and Burkina Faso confirm similar species compositions, underscoring both the adaptability of *Ae. aegypti* and the invasive expansion of *Ae. albopictus* across West and Central Africa [35,36].

This study provides updated and complementary information to previous investigations on *Aedes* populations in Benin [10,11,12], which mainly focused on urban vector distribution or density. Unlike earlier works, our integrated approach encompassed multiple urban and peri-urban municipalities with high epidemic potential. This broader scope revealed marked differences in the typology, frequency, and productivity of breeding sites, demonstrating how *Aedes* larval habitats have diversified in recent years. The growing dominance of plastic containers, tyres, and discarded items reflects ongoing ecological adaptation of *Aedes* mosquitoes to environmental changes and waste management practices. This trend is particularly evident in *Ae. albopictus*, whose geographical distribution appears to be expanding into areas where the species had not previously been reported.

The detection of *Ae. albopictus* in certain municipalities likely signals its recent establishment in peri-urban areas, possibly facilitated by interregional trade and population mobility. The coexistence of *Ae. albopictus* and *Ae. aegypti* represents a notable ecological shift that could amplify arbovirus transmission potential. Their simultaneous occurrence in domestic habitats, particularly during wet seasons, may sustain continuous viral circulation. Combined with the higher overall vector densities observed compared to earlier studies [11,12], this ecological overlap suggests a potentially greater epidemic risk than previously recognized.

Most larval habitats identified were artificial or domestic containers, jars, buckets, jerrycans, and discarded tyres, which are widely recognized as the most productive breeding sites for *Aedes* mosquitoes in Africa. These habitats are sustained by domestic water-storage practices and poor waste management [33,37,38]. Discarded tyres, in particular, remain year-round breeding hotspots due to their capacity to collect rainwater and organic matter, a pattern consistent with observations globally [39,40]. The heavy reliance on artificial containers highlights the anthropogenic factors driving arbovirus risk in the study area.

Both indoor and outdoor breeding sites were found. Indoor containers (e.g., jars and jerrycans) enhance household-level transmission potential, while outdoor sites (e.g., tyres, barrels) act as neighborhood-scale reservoirs of *Aedes* populations. Studies from West Africa and Latin America report higher productivity in outdoor habitats due to reduced maintenance and greater rainwater accumulation [37,41]. These findings indicate that effective vector control should combine household-level measures (covering and maintaining water containers) with broader community actions such as proper waste disposal and tyre management [42,43].

Significant differences in entomological indices (HI, CI, and BI) among municipalities reflect local ecological and socio-economic variations. These differences can be attributed to factors such as population density, domestic water management, types of storage containers used, and local microclimatic conditions. Highly urbanized municipalities, characterized by dense populations, frequent water shortages, and abundant uncovered containers, recorded the highest indices. These findings are consistent with reports from Nigeria and Ghana, where population density and poor water-storage practices significantly increase *Aedes* reproductive potential [44,45]. Climatic variability, household behaviour, and economic activities such as tyre repair workshops are also known to shape these patterns [46].

All three indices (CI, BI, and HI) exceeded WHO thresholds for epidemic risk [47,48], indicating a substantial potential for dengue, chikungunya, Zika, and yellow fever outbreaks. Similar high indices have been reported in Abidjan and Benin, emphasizing that West African cities remain highly vulnerable to explosive arboviral epidemics [12,35]. The high *Aedes* densities and widespread breeding habitats place Benin at continuous risk of arboviral epidemics [49]. Moreover, overlapping symptoms between dengue and malaria complicate clinical diagnosis and delay outbreak detection.

Given the increasing reports of arbovirus co-circulation in West Africa, there is an urgent need to strengthen diagnostic capacity, integrate entomological and virological surveillance, and establish early-warning systems in Benin. Control efforts should target the most productive breeding habitats, jars, buckets, tyres, and jerrycans, through environmental sanitation, container management, and improved water-storage practices [37,41]. Community engagement remains essential, as demonstrated in Nigeria, where participatory approaches improved the sustainability of interventions. Integrated Vector Management (IVM), linking arbovirus control with malaria and filariasis programmes, offers a cost-effective and synergistic framework [43].

While this study provides current insights into the diversity and dynamics of *Aedes* breeding sites in Benin, certain methodological limitations must be acknowledged. The cross-sectional design captures only a single time point and may not fully account for seasonal variation, a critical factor given rainfall-driven fluctuations in *Aedes* breeding [46,50]. Moreover, the limited number of surveyed households per commune may restrict the generalizability of exact indices.

Future studies should therefore adopt longitudinal designs to capture seasonal changes in vector dynamics. Integrating entomological and virological surveillance, such as screening *Aedes* pools for arboviruses using RT-qPCR and sequencing, will be crucial. Strengthening laboratory and field surveillance capacity will enhance arbovirus detection and improve outbreak preparedness in Benin and across the subregion. Despite these limitations, this study significantly advances understanding of arbovirus vector ecology in Benin and provides a robust foundation for guiding future surveillance and vector-control initiatives.

## 5. Conclusions

The present study highlights the predominance and diversity of artificial breeding sites supporting the pre-imaginal stages of *Aedes* mosquitoes in domestic and peri-domestic environments. These habitats provide highly favourable conditions for mosquito development, thereby increasing the potential risk of arboviral disease transmission, particularly dengue. The consistently high epidemic risk indices underscore the urgent need for an integrated vector management approach combining environmental sanitation, targeted community engagement, and sustained public health education. Importantly, these findings provide valuable baseline data to guide the National Neglected Tropical Diseases Control Programme in optimizing strategies for arbovirus prevention and control in Benin.

## Figures and Tables

**Table 1:** Mosquito species composition per type of breeding sites.

Categories of Breeding Sites	Breeding Sites	N of Emerged Mosquitoes	*Ae. Aegypti* (%)	*Ae. albopictus* (%)	*Ae. vittatus* (%)	*An. gambiae* (%)	*Cx. nebulosus* (%)	*Cx. tigripes* (%)	*Cx. quinquefasciatus* (%)	*p* Value
Domestic containers	Drinking troughs	2	100	-	-	-	-	-	-	-
	jerrycans	287	83.97 ^a^	1.05 ^b^	2.79 ^b^	0.70 ^b^		1.05 ^b^	10.45 ^c^	<0.0001
	Kettle	11	100	-	-	-	-	-	-	-
	Canaries	57	91.23 ^a^	-	-	-	-	-	8.77 ^b^	<0.0001
	Jar	436	71.33 ^a^	-	0.69 ^b^	3.21 ^b^	0.46 ^b^	1.83 ^b^	22.48 ^c^	<0.0001
	Bucket	342	86.55 ^a^	0.58 ^b^	-	0.58 ^b^	-	-	12.28 ^c^	<0.0001
	Cistern	126	72.22 ^a^	-	-	2.38 ^b^	-	-	25.4 ^c^	<0.0001
	Drum	162	91.36 ^a^	-	-	1.85 ^b^	-	-	6.79 ^c^	<0.0001
Discarded container	Tin can	128	50 ^a^	-	-	0.78 ^b^	-	0.78 ^b^	48.44 ^a^	<0.0001
Tyres	Tyres	394	41.11 ^a^	0.25 ^b^	2.79 ^b^	1.02 ^b^	1.02 ^b^	0.5 ^b^	53.3 ^c^	<0.0001
Other breeding sites	Mortar	43	62.79 ^a^	-	-	-	9.30 ^b^	-	27.91 ^c^	<0.0001

N: number, The letters a, b, and c indicate that the proportions of species observed in breeding sites are significantly different (*p* = 0.0001).

**Table 2:** Results of the χ^2^ test for the different categories of breeding sites with *p*-values.

	Position	Discarded Containers	Domestic Containers	Naturel Containers	Others	Tyres	*p* Value
potential breeding sites	Indoor	0.32 ^c^	85.46 ^a^	0	0.53 ^c^	13.7 ^b^	<0.0001
	Outdoor	2.43 ^c^	56.31 ^a^	0.97 ^c^	1.94 ^c^	38.35 ^b^	<0.0001
Aedes positive breeding sites	Indoor	0.72 ^c^	83.86 ^a^	0	0.72 ^c^	14.7 ^b^	<0.0001
	Outdoor	3.7 ^b^	41.98 ^a^	0	0.62 ^b^	53.7 ^a^	<0.0001

Letters a, b, and c indicate that the proportions of habitats are significantly different (*p* = 0.0001).

**Table 3:** Assessment of the risk of an arboviral disease epidemic using the House, Container, and Breteau indices.

Commune	N Breeding Sites	Positive Breeding Sites	N Houses	Positive Houses	House Index 95% CI	Risk Level	Container Index 95% CI	Risk Level	Breteau Index 95% CI	Risk Level
Bante	181	87	37	20	54.05 [38.4–69]	High	48.06 [40.9–55.3]	High	235.1 [185.7–284.5]	High
Bassila	122	80	35	19	54.28 [38.2–69.5]	High	65.57 [56.8–73.4]	High	228.6 [178.5–278.7]	High
Bohicon	39	33	12	8	66.66 [39.1–86.2]	High	84.61 [70.3–92.8]	High	275 [181.2–368.8]	High
Comé	46	22	10	9	90 [59.6–98.2]	High	47.82 [34.1–61.9]	High	220 [128.1–311.9]	High
Dassa-Zounmé	60	55	24	10	41.66 [24.5–61.2]	High	91.66 [81.9–96.4]	High	229.2 [168.6–289.7]	High
Djougou	60	47	37	10	27.02 [15.4–43]	Medium	78.33 [66.4–86.9]	High	127 [90.7–163.3]	High
Grand Popo	29	12	20	7	35 [18.1–56.7]	Medium	41.37 [25.5–59.3]	High	60 [26.1–93.9]	High
Savalou	142	85	38	19	50 [34.8–65.2]	High	59.85 [51.6–67.6]	High	223.7 [176.1–271.2]	High
Savè	192	86	53	24	45.28 [32.7–58.5]	High	44.79 [37.9–51.9]	High	162.3 [128–196.6]	High

## Data Availability

The original contributions presented in this study are included in the article. Further inquiries can be directed to the corresponding authors.

## Abbreviations

Ae	*Aedes*
An.	*Anopheles*
Cx	*Culex*
CREC	Centre de Recherche Entomologique de Cotonou
WHO	World Health Organization
RT-qPCR	Reverse Transcription quantitative Polymerase Chain Reaction
